# Mass Spectrometry Imaging of Lipid and Metabolite Distributions in Cysts of *Besnoitia besnoiti*-Infected Bovine Skin

**DOI:** 10.1021/jasms.4c00466

**Published:** 2025-04-08

**Authors:** Katja R. Wiedemann, Stefanie Gerbig, Parviz Ghezellou, Alejandra Pilgram, Carlos Hermosilla, Anja Taubert, Liliana M. R. Silva, Bernhard Spengler

**Affiliations:** † Institute of Inorganic and Analytical Chemistry, Justus Liebig University Giessen, 35392 Giessen, Germany; ‡ Institute of Parasitology, Justus Liebig University Giessen, 35392 Giessen, Germany; § Egas Moniz Center for Interdisciplinary Research (CiiEM), Egas Moniz School of Health & Science, 2829-511 Caparica, Almada, Portugal; ∥ MED − Mediterranean Institute for Agriculture, Environment and Development & CHANGE − Global Change and Sustainability Institute, Universidade de Évora, 7006-554 Évora, Portugal

**Keywords:** Besnoitiosis, AP-SMALDI, Ultrahigh-resolution mass spectrometry imaging, Host−parasite interaction, *Besnoitia besnoiti*, Apicomplexa

## Abstract

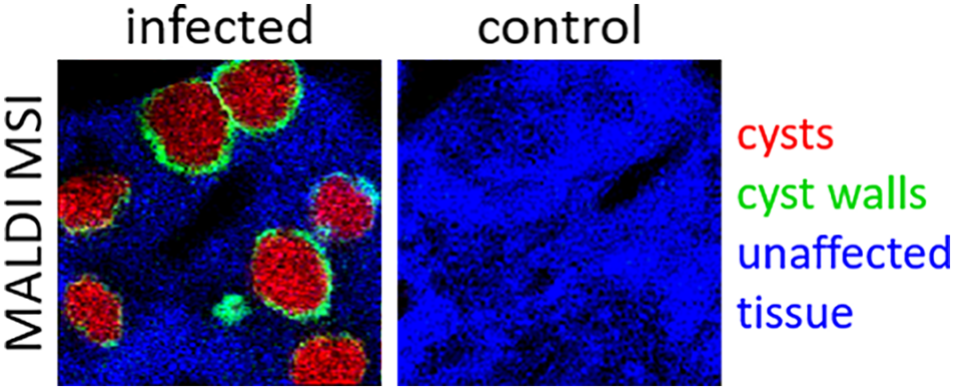

Bovine besnoitiosis is a disease caused by the obligate intracellular parasite *Besnoitia besnoiti*. During its chronic stage, the parasite forms large, thick-walled cysts of up to 600 μm in diameter in the skin and other tissues. To assess an overview of parasite-induced metabolic changes during chronic infection, *B. besnoiti*-infected skin samples were analyzed by high-resolution atmospheric-pressure scanning microprobe matrix-assisted laser desorption/ionization mass spectrometry imaging (AP-SMALDI MSI). Overall, infection-driven, significant changes of 467 lipids and metabolites were found in comparison to noninfected control samples. Most of them belong to the group of phosphatidic acids (PAs), phosphatidylserines (PSs), phosphatidylcholines (PCs)/phosphatidylethanolamines (PEs), triacylglycerides (TGs), phosphatidylinositols (PIs) and phosphatidylglycerols (PGs). When these quantitative data were combined with analyses on the lateral distribution of respective infection markers, MS images of significantly changed ion signals with specific lateral distributions were generated, matching with typical biological structures as observed in Hematoxylin and eosin (H&E)-stained tissue sections. Ultrahigh-resolution MALDI MSI with a pixel size of 2 μm and 3-dimensional reconstruction gave further insights into cyst construction.

## Introduction

For 30 years, matrix-assisted laser desorption/ionization mass spectrometry imaging (MALDI MSI) has been an established method for the (untargeted) analysis of biological tissue while maintaining the topological information on the sample.^1^ The matrix-coated sample is analyzed in a rasterized fashion, which allows for the coregistration of MS spectra and the corresponding laser spot coordinates. The matrix is carefully selected based on acidic/basic properties, crystal size, and analyte solubility.

Over the recent years, MALDI MSI instrumentation has significantly improved, now achieving a lateral resolution below 2 μm^2^ in dedicated workflows. Furthermore, mass analyzers based on orbital trapping or ion cyclotron resonance (ICR) provide accurate *m*/*z* determination with mass errors below ±1 ppm when applying adequate internal calibration measures.^3,4^ Therefore, natural compounds in biological specimens can be assigned based on elemental composition and, in the case of sufficient signal intensity, can also be structurally characterized using on-tissue MS/MS.

Using new software approaches, several mass spectrometry data sets being recorded on adjacent tissue sections can be stitched together, resulting in 3-dimensional MS images, reflecting structures of interest.^5^

*Besnoitia besnoiti* is a cyst-forming and obligate intracellular apicomplexan parasite that causes bovine besnoitiosis, a chronic and debilitating disease manifested by cutaneous and systemic alterations in cattle.^6,7^ Bovine besnoitiosis is endemic in Asia and Africa and re-emerging in European countries.^8−13^ Besnoitiosis significantly impacts the individual welfare of infected bovine and causes considerable financial losses in the cattle industry,^13,14^ especially because it can cause sterility in infected bulls.^14,15^. Besnoitiosis includes subacute, acute, and chronic phases.^16^ Outbreaks of cattle besnoitiosis are characterized by nonspecific symptoms such as fever in the acute phase and typical clinical signs such as severe skin alterations or scleroderma in the chronic phase of the disease. As a relevant consequence of the disease, infections of male reproductive tissues (e.g., testicles) may result in bull sterility.^17,18^ Acute infection is characterized by the presence of fast-proliferating tachyzoites that mainly replicate in vascular endothelial cells, causing vascular lesions. In contrast, in the chronic phases of besnoitiosis, slow-replicating bradyzoites proliferate in mesenchymal cells forming large thick-walled tissue cysts mainly in dermis, sclera, and mucosa.^19^ Due to their large size, tissue cysts are macroscopically detectable and, when being localized in the sclera or vaginal mucosa, may even serve for inspective diagnostics in living animals.^19^

*B. besnoiti* tissue cysts are large and show an average diameter of 200 μm. Cystic tissues often present pericystic inflammatory reactions, depending on the duration of infection and the affected tissue type.^20^ Mature cysts can reach 600 μm in diameter.^16,19^ Hence, these tissue cysts are easily demonstrated in skin sections of infected cattle experiencing the chronic phase of disease.^16^ Tissue cysts consist of a hypertrophied host cell with enlarged nuclei, an intracytoplasmic parasitophorous vacuole (PV) with bradyzoites, a sometimes vacuolated inner cyst wall, and an outer cyst wall (outermost acellular layer) in more developed/mature cysts.^16,21^ The outer cyst wall comprises multiple layers of collagen fibrils, arranged in a circular way most probably collagen type I fibers, while the inner cyst wall is made up of elements of the extracellular matrix.^16^ Cysts contain only a small rim of host cell cytoplasm, which surrounds the PV, and present a hypertrophic host cell nuclei at their periphery.^16,22^

Bradyzoites typically have a diameter of approximately 2 μm and are 7.5 μm long. Currently, morphological aspects of cysts containing bradyzoites have been described in detail,^19,21,23^ and there is also reported data on the proteome of the different life stages of *B. besnoiti*.^24^ Moreover, some data on relevant *B. besnoiti* tachyzoite-driven changes of key metabolic pathways and selected metabolites have been reported,^25,26^ e.g., transcriptomic data showing altered pathways related to lipid metabolism in bradyzoites.^19^

To date, serological tests for besnoitiosis diagnostics are established,^10,27−29^ but major knowledge of parasite-driven host cell alterations or even of major steps of the life cycle (currently unknown definitive hosts) is still lacking.^12^ Additionally, neither treatments nor licensed vaccines are currently available in Europe.^12^

MALDI MSI has been used in various cases to study parasites^30−34^ and host–parasite interactions.^35−37^ Therefore, it was the method of choice to gain further insights into *Besnoitia besnoiti* bradyzoite cysts and their host–parasite interactions, especially in the field of lipidomics.

## Materials and Methods

### Chemicals

A list of all chemicals used can be found in Table S1.

### Tissue Samples

Natural *B. besnoiti* infection was confirmed via a polymerase chain reaction investigation of a suspected infected cow from the South of France. The animal was euthanized due to severe clinical conditions. At necropsy, bovine besnoitiosis in the scleroderma phase was confirmed as multiple whitish punctuated cysts were observed in sclera and in mucocutaneous junctions of the mouth and anus. Skin biopsies were collected from the neck, elbow, and shoulder regions. Skin samples were maintained at 4 °C and immediately sent to the Institute of Parasitology at Justus Liebig University Giessen, where they were conserved frozen at −80 °C until further analysis.

For noninfected control samples, neck skin samples were collected from the local abattoir near Giessen, Germany, from cows originating in farms without any history of besnoitiosis and were treated as previously stated.

### Sample Preparation

Before sample preparation, hair was removed from skin samples, and sections of 20 μm thickness from different parts of the sample (see Figure 2) were prepared at −25 °C using a Cryostat Microm HM 525 (Thermo Fisher Scientific, Dreieich, Germany). Sections were thaw-mounted onto glass slides. Microscopic images were recorded with a digital microscope (VHX-5000, Keyence, Neu-Isenburg, Germany) before matrix application and after staining.

For 3D-imaging experiments, smaller pieces of the skin samples were embedded in 10% gelatin solution. After the samples were frozen, consecutive sections of 14 μm thickness were prepared as previously stated.

Matrix application was performed with an ultrafine pneumatic sprayer (SMALDIPrep, TransMIT GmbH, Giessen, Germany) as described elsewhere.^38^ For positive-ion mode, 100 μL of 2,5-dihydroxybenzoic acid (DHB) solution (30 mg/mL, acetone/H_2_O/trifluoroacetic acid (49.95:49.95:0.1, *v*:*v*:*v*)) was applied. Flow rate was set to 10 μL/min, and nitrogen pressure was set to 1 bar. For negative-ion mode, 400 μL of 1,5-diaminonaphthalene (DAN) solution (3.3 mg/mL, H_2_O/methanol (0.1:0.9, *v*:*v*)) was applied with a flow rate of 30 μL/min.

### Atmospheric-Pressure Scanning Microprobe Matrix-Assisted Laser Desorption/Ionization Mass Spectrometry Imaging (AP-SMALDI MSI) Analysis

For AP-SMALDI MSI analyses, an orbital trapping mass spectrometer (Q Exactive HF, Thermo Fisher Scientific, Bremen, Germany) was used in combination with a high-resolution MS imaging ion source (AP-SMALDI^5^ AF, TransMIT GmbH, Giessen, Germany). Instrumental settings are described in Table S2. Pixel size was set to 5 μm. The instrument was freshly calibrated prior to each measurement. Therefore, a blank glass slide spray coated with DHB solution was used. Matrix-cluster ions used for mass calibration are listed in Table S2. Measurements were performed in triplicate for each sample type (infected neck, infected shoulder, infected elbow, control1, control2).

### Ultrahigh-Resolution AP-SMALDI MSI Analysis

For ultrahigh-resolution experiments (2 μm pixel size), a prototype ion-source from TransMIT, coupled to a Q Exactive mass spectrometer, was used. The same settings as those for higher pixel sizes were chosen.

### On Tissue MS/MS Analysis

On tissue MS/MS experiments were performed with the same instrument settings on an Orbitrap Exploris 480 mass spectrometer (Thermo Fisher Scientific, Bremen, Germany) equipped with an AP-SMALDI^5^ AF ion source. To achieve sufficient signal intensity, pixel sizes were increased from 5 to 20 μm and the so-called full-pixel mode was used to ablate the whole pixel area. Ions were chosen after statistical processing (see below) and loaded via an inclusion list into the method. Top 5 ions were fragmentated, and the normalized collision energy was set to 20.

### Hematoxylin and Eosin (H&E) Staining

Tissue sections were rinsed with ethanol to wash off the matrix. Afterward, sections were gradually rehydrated in 100%, 70%, and 40% ethanol and deionized water (2 min, each). Then, samples were stained with hematoxylin solution for 12 min, blued for 10 min in tap water, and washed in deionized water for 5 min. After 1 min of incubation in eosin y solution, samples were dehydrated in deionized water, 40%, 70%, and 100% ethanol and xylene for 2 min each. Finally, samples were covered with Eukitt and a glass coverslip. H&E staining was used for infection confirmation.

### Data Analysis

For quick visualization and annotation, data were uploaded to Metaspace.^39^ All annotated signals (FDR = 10%, found in at least one of these databases: HMDB,^40^ Lipid Maps,^41^ SwissLipids,^42^ Core Metabolome Database) were exported and used to compute summed signal intensity lists with Mirion.^43^ Subsequently, summed signal intensities of the three groups (infected, control1, and control2) were compared using MetaboAnalyst.^44^ No filtering was applied. Data were normalized row-wise to a constant sum and transformed by log10 normalization. Then, principal component analysis (PCA) was performed. In order to find statistically significant differences, analysis of variance (ANOVA) with *p* ≤ 0.05 followed by Tukey’s post hoc test was conducted.

### MS Image Generation

Infection markers, significantly altered in signal intensities, were selected to create MS images with Mirion. Respective distribution patterns were examined manually, and red-green-blue (RGB) overlay images were created.

### 3D Reconstruction

For three-dimensional reconstruction, measurements of 28 consecutive tissue sections were performed in positive-ion mode on the same instrument with the same settings as those for the 5 μm pixel size experiments. However, according to a section thickness of 14 μm, a pixel size of 14 μm was chosen, creating cubic voxels. Each measurement consisted of 50 × 50 pixels, leading to an analyzed area of 700 × 700 μm^2^ and being large enough to contain several cysts while keeping the measurement time in an affordable scope. All data were loaded into M2aia software.^4^ According to previous measurements, the ion signal at *m*/*z* 824.56, annotated as either phosphatidylethanolamine (PE) [PE P-42:7 + Na]^+^ or more probably phosphatidylcholine (PC) [PC 36:2 + K]^+^, was chosen for cyst visualization because it was found in the whole tissue but with higher intensities inside the cysts. After TIC normalization, all 28 individual ion images were created and stacked as well as aligned in M2aia, leading to a three-dimensional reconstruction.

### Parasite Stage Comparison

Lipids and metabolites, significantly altered in signal intensities due to the presence of *B. besnoiti* bradyzoites, were compared with tachyzoite markers derived from an *in vitro* study, described in a previous publication.^34^ Measurements from three tachyzoite replicates were stitched using Mirion and uploaded to Metaspace. Since the tachyzoite samples consisted of isolated parasites, all detected signals were regarded as marker signals for tachyzoites. A list containing all annotations (FDR = 10%, HMDB) was generated and compared manually with the bradyzoites markers.

## Results and Discussion

### Visualization of Large Intradermal *B. besnoiti*-Tissue Cysts

**Figure 1:**
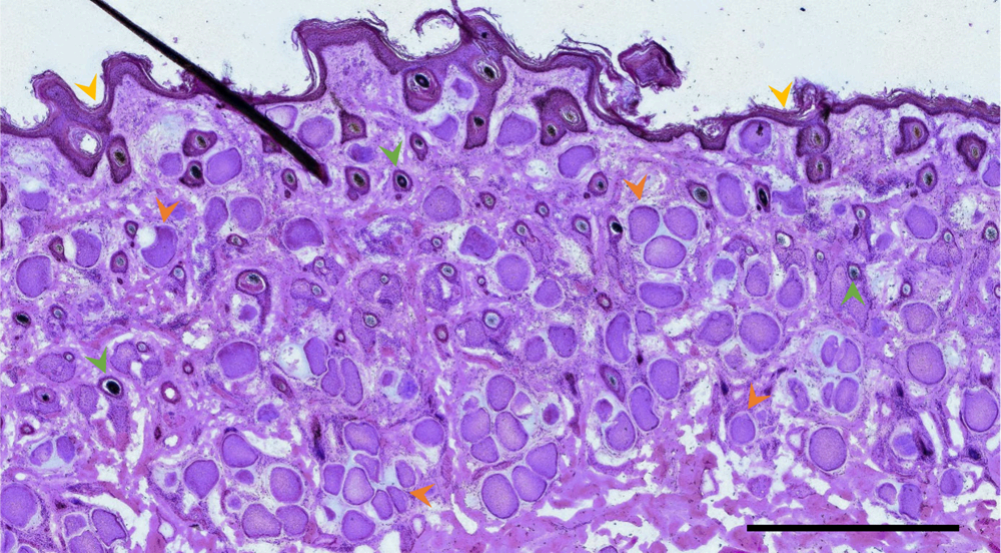
H&E-stained freshly prepared cryosection. *Besnoitia besnoiti* bradyzoite-containing skin cysts of different sizes and shapes are easily recognizable (examples indicated by orange arrowheads). Other relevant structures, used for comparison of optical and corresponding MS images, are hair follicles (examples indicated by green arrowheads) and the epidermis (yellow arrowheads). Scale bar is 1 mm.

Based on an average diameter of 200 μm,^20^ most intradermal cysts are macroscopically visible without any further visual aids. In the current study, skin cryosections of naturally *B. besnoiti*-infected cattle were H&E stained prior to any further analysis to assess cyst burden and quality. As illustrated in Figure 1, this animal was severely infected with *B. besnoiti* tissue cysts which were highly abundant in the skin sample, remained intact, and did not show any artifactual loss of content due to sample processing or tissue degeneration. H&E staining also verified that the two control animals showed no tissue cysts (see Figure S1) and were noninfected with *B. besnoiti*.

**Figure 2:**
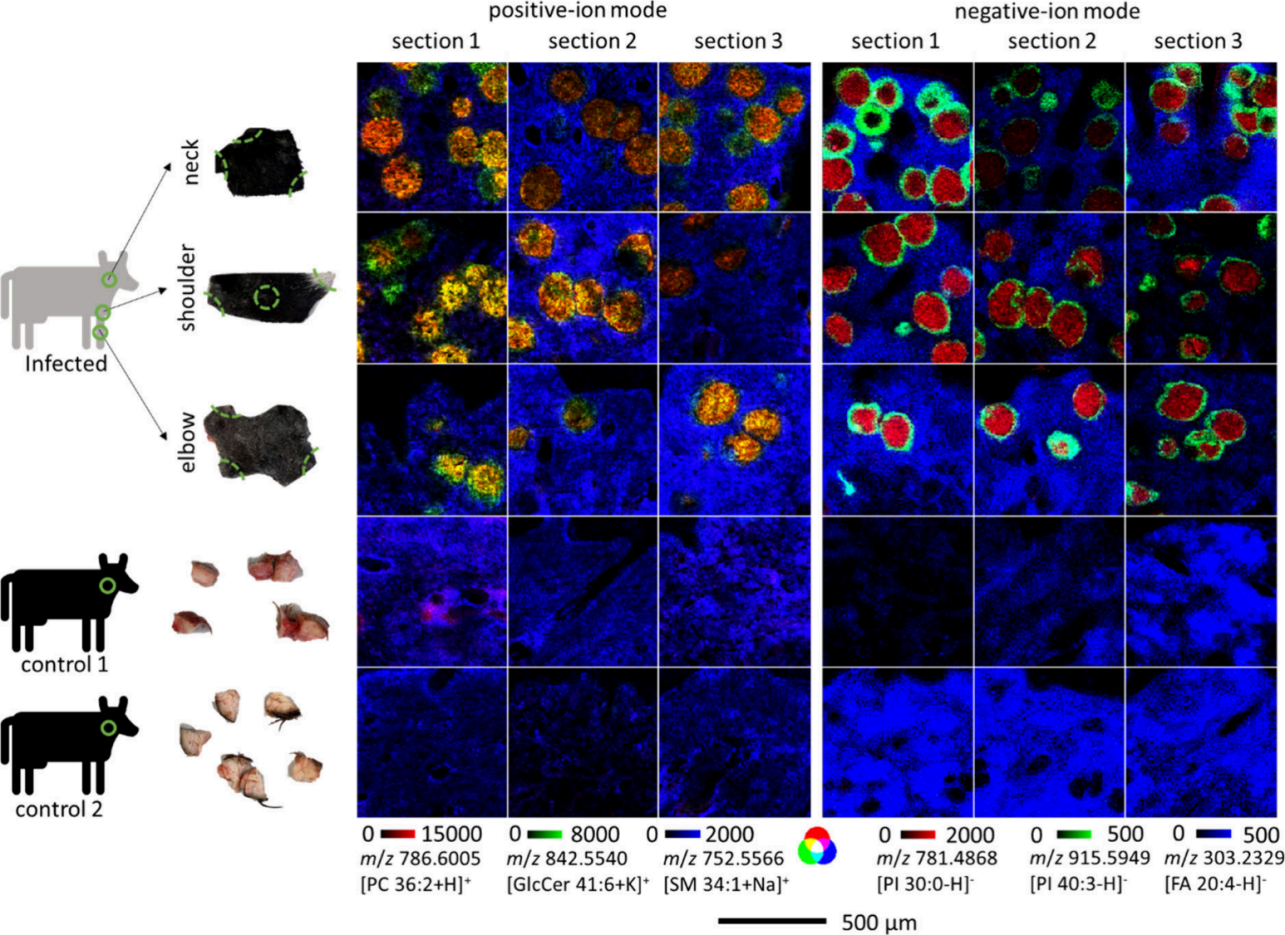
Scheme of the sample origin and resulting ion images of selected markers. *B. besnoiti*-infected samples originated from three different parts of one infected animal: the neck, shoulder, and elbow. Cryosections of 20 μm thickness were prepared from three different sections per skin part, as illustrated with dotted green lines. For comparison, neck samples from two healthy control animals were analyzed in the same manner. MS experiments were performed in positive- and negative-ion mode. For positive-ion mode, *m*/*z* 786.6005, annotated as PC 36:2, [C_44_H_84_NO_8_P + H]^+^, shown in red, *m*/*z* 842.5540, annotated as GlcCer 41:6, [C_47_H_81_NO_9_ + K]^+^, shown in green, and *m*/*z* 725.5566, annotated as SM 34:1, [C_39_H_79_N_2_O_6_P + Na]^+^, shown in blue, were overlaid. For negative-ion mode, *m*/*z* 781.4868, annotated as PI 30:0, [C_39_H_75_O_13_P – H]^−^, shown in red, *m*/*z* 915.5949, annotated as PI 40:3, [C_49_H_89_O_13_P – H]^−^, shown in green, and *m*/*z* 303.2329, annotated as FA 20:4, [C_20_H_32_O_2_ – H]^−^, shown in blue, were overlaid. Intensities were adjusted to the same intensity levels for all of the MS images shown. Scale bar applies to all MS images.

We used skin samples from three different parts of one naturally *B. besnoiti*-infected animal: from the neck, the shoulder, and the elbow (see Figure 2). While the neck and shoulder samples had a high cyst burden, only a few cysts were found in the elbow sections. Results among these three groups were comparable. This is reasonable because they all originated from the same animal, allowing for comparison between high and low cyst-burden areas.

MSI analysis of *B. besnoiti*-infected bovine skin tissue and statistical analysis of signal intensities in comparison to control tissue revealed several infection-induced changes with statistical significance. PCA plot and dendrogram can be found in Figure S2. Since MSI preserves the spatial information, detected ions can be attributed to either host tissue, cyst walls, or cyst content. Overall, 552 ions affected by *B. besnoiti* infection were found, 273 of which were detected in positive-ion mode and 279, in negative-ion mode. However, some species were found in both positive- and negative-ion modes due to adduct formation. For example, LysoPE (18:1) contributed four times to the total number of relevant ions: it was found in positive-ion mode as +H^+^, +K^+^ and +Na^+^ adducts and additionally in negative-ion mode as a deprotonated ion. In total, 36 molecules were detected as several positive adducts, and 23 molecules were detected in positive- as well as negative-ion mode. By correcting this artifact, a total of 467 unique compounds were found that are influenced by parasitic infection, with most of them being lipids. We analyzed all 552 ions without further differentiation of adduct-related duplicates. A list of all annotated analytes can be found in an additional data sheet in the Supporting Information (color coding: green, individual compounds; yellow, adduct- or both-ion-mode-related duplicates); few examples with interesting lateral distributions are in Table 1. There were no statistically significant differences in the lipid level between the infected samples from three different parts of the animal. With on-tissue MS/MS, we were able to identify 18 ions, 9 in positive- and 9 in negative-ion mode, by detecting at least the headgroup of the phospholipid, enabling the differentiation between isomeric phospholipids such as PCs and PEs. An example can be seen in Figure S3, identifying *m*/*z* 671.4654 as phosphatidic acid (PA) (16:0_18:2). However, potential in-source fragmentation cannot be excluded. Therefore, the headgroup might have also lost an ethanolamine or serine group prior to intentional fragmentation. For corresponding fragment ions, see Table S3.

**Table 1:** Examples of Ions Found with Significantly Different Signal Intensities When Comparing Infected Samples with Control Samples with Characteristic Lateral Distributions

exemp. annotation	mol. class	formula	add.	*m*/*z* meas.	structure	MS/MS	found in Tachyzoites?
Glucose phosphate	COH	C_6_H_13_O_9_P	[M – H]^−^	259.0224	higher in cysts	phosphate group	
FA(18:1)	FA	C_18_H_34_O_2_	[M – H]^−^	281.2486	higher in cysts		
Acetylglucosamine sulfate	COH	C_8_H_15_NO_9_S	[M – H]^−^	300.0395	walls		
FA(20:4)	FA	C_20_H_32_O_2_	[M – H]^−^	303.2330	surrounding tissue		
Fructose bisphosphate	COH	C_6_H_14_O_12_P_2_	[M – H]^−^	338.9888	cysts		
Oleoylcarnitine	carnitine	C_25_H_47_NO_4_	[M + H]^+^	426.3578	higher in walls		
LysoPC(18:1)	LysoPC	C_26_H_52_NO_7_P	[M + H]^+^	522.3554	higher in cysts		yes
DG(14:0_18:1_0:0)	DG	C_35_H_66_O_5_	[M + K]^+^	605.4542	cysts		
SM(d18:1_14:0)	SM	C_37_H_75_N_2_O_6_P	[M + H]^+^	675.5436	surrounding tissue		
PC(14:0_18:1)	PC	C_40_H_78_NO_8_P	[M + H]^+^	732.5538	higher in cysts	PC 32:1	yes
PC(14:0_20:1)	PC	C_42_H_82_NO_8_P	[M + H]^+^	760.5851	higher in cysts	PC 34:1	yes
PI(16:0_14:0)	PI	C_39_H_75_O_13_P	[M – H]^−^	781.4873	cysts	Kadesch: PI	
PC(18:1_18:1)	PC	C_44_H_84_NO_8_P	[M + H]^+^	786.6007	higher in cysts	PC 36:2	yes
PC(14:0_20:1)	PC	C_42_H_82_NO_8_P	[M + K]^+^	798.5410	higher in walls	PC 34:1	
PC(18:1_18:1)	PC	C_44_H_84_NO_8_P	[M + K]^+^	824.5566	higher in cysts	PC 36:2	
GlcCer(iso-t17:0_24:6)	GlcCer	C_47_H_81_NO_9_	[M + K]^+^	842.5543	higher in walls		
PI(18:3_22:0)	PI	C_49_H_89_O_13_P	[M – H]^−^	915.5968	walls	Kadesch: PI	

Interpretation of our data is challenging since little is known about *B. besnoiti* infection and host–parasite interaction on the lipidomic and metabolic level. However, other parasites from the same family Sarcocystidae (e.g., *Toxoplasma gondii* and *Neospora caninum*) and the same subphylum Apicomplexa (e.g., *Plasmodium falciparum*) that have been studied in more detail can serve as a basis for careful speculation. It is well-known that apicomplexan parasites scavenge several molecular classes from their host cells for intracellular development. For example, Apicomplexa are generally considered as defective in cholesterol synthesis and have to scavenge cholesterol from their host cells for successful replication.^45^ Consequently, most of the altered lipids identified here likely originated from the host and were not produced by the parasite itself. Due to its obligatory intracellular lifestyle within a PV and enclosed by a cyst wall, the parasite has no direct access to extracellular molecule sources and therefore satisfies its need from the host cell.^46,47^ Besides other molecule classes, apicomplexan parasites scavenge host lipids, as reported for phospholipids, fatty acids, and cholesterol in *T. gondii*.^48,49^ Likewise, *B. besnoiti* tachyzoites were recently shown to drive host cellular cholesterol biosynthesis and to profit from enhanced availability of exogenous lipid sources.^47^ Therefore, we here expected to detect parasite-infection-driven changes in lipid signals. On the other hand, related apicomplexan parasites like *T. gondii* and *P. falciparum* synthesize PCs by themselves during intracellular development,^50,51^ which aligns well with our results since several PCs were found inside the parasitic cysts.

Figure 2 nicely reflects that we were able to visualize *B. besnoiti*-formed cysts inside the tissue using AP-SMALDI MSI. By using simple statistics, we also found differences in signal intensities: Except for the sphingomyelin (SM) 34:1 (blue ion channel in positive-ion mode), all ions shown in Figure 2 were found to have significant intensity changes when comparing infected samples to controls. However, results can only be tentative, since only one infected animal was studied (*n* = 1), as it is extremely difficult to access fresh samples from infected animals in Germany. Cyst burden was different in the three regions (higher cyst load in the neck and shoulder samples than in the elbow sample), but there were no significant differences in the lipid level between these three groups.

**Figure 3:**
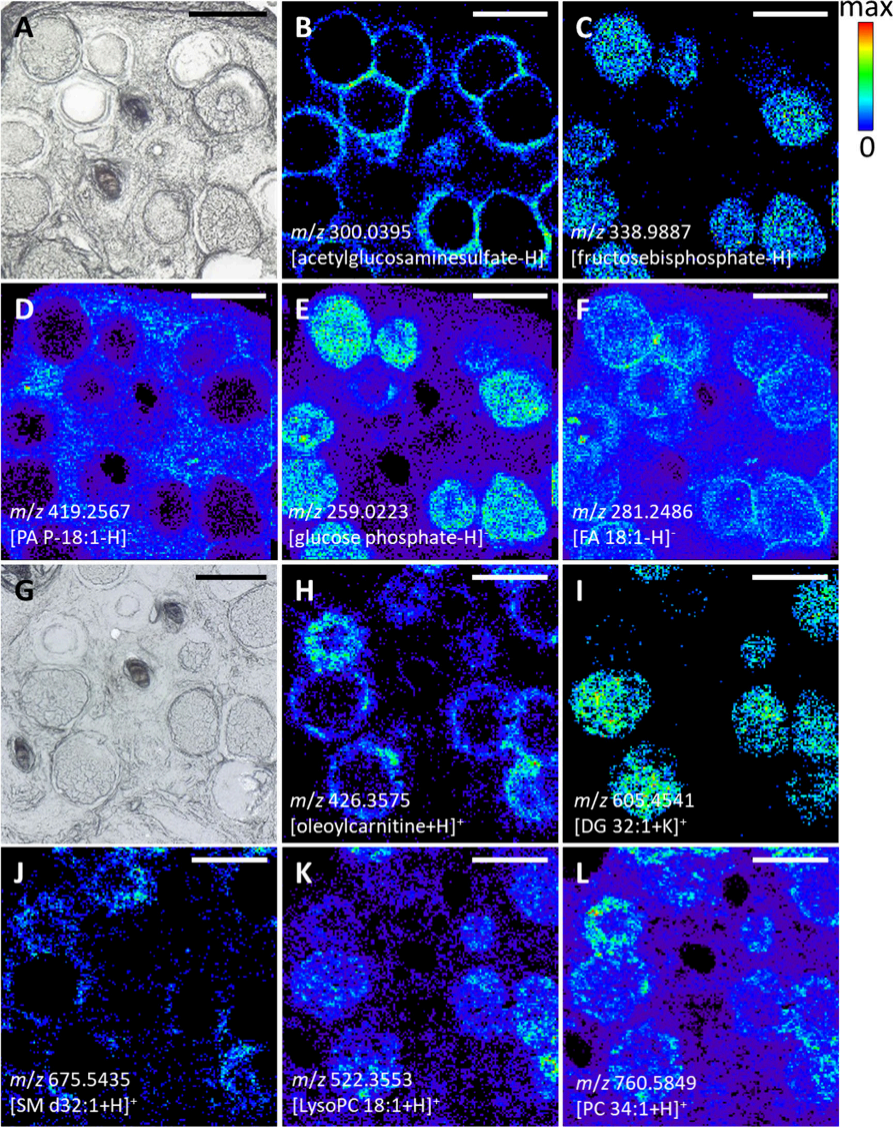
Morphological structures induced by *B. besnoiti* parasite infection, as represented by selected ion signals in the MS images in comparison with the microscopic images. (A, G) Corresponding light microscopic images taken prior to the measurements. (B) *m*/*z* 300.0395, annotated as acetylglucosaminesulfate, [C_8_H_15_NO_9_S – H]^−^, representing the walls of the parasite-induced cysts; (C) *m*/*z* 338.9887, annotated as fructosebisphosphate, [C_6_H_14_O_12_P_2_ – H]^−^, representing the contents of the parasite-induced cysts; (D) *m*/*z* 419.2567, annotated as PA P-18:1, [C_21_H_41_O_6_P – H]^−^, being present in the whole tissue section but depleted inside the cysts; (E) *m*/*z* 259.0223, annotated as glucose phosphate, [C_6_H_13_O_9_P – H]^−^, being present in the whole tissue section but enriched inside the cysts; (F) *m*/*z* 281.2486, annotated as FA 18:1, [C_18_H_34_O_2_ – H]^−^, being present in the whole tissue section but enriched in the cyst walls; (H) *m*/*z* 426.3575, annotated as oleoylcarnitine, [C_22_H_44_NO_6_P + H]^+^, representing the walls of the parasite-induced cysts; (I) *m*/*z* 605.4541, annotated as DG 32:1, [C_35_H_66_O_5_ + K]^+^, representing the content of the parasite-induced cysts; (J) *m*/*z* 675.5435, annotated as SM d32:1, [C_37_H_75_N_2_O_6_P + H]^+^, being present in the whole tissue section but depleted inside the cysts; (K) *m*/*z* 522.3553, annotated as LysoPC 18:1, [C_26_H_52_NO_7_P + H]^+^, being present in the whole tissue section but enriched inside the cysts; (L) *m*/*z* 760.5849, annotated as PC 34:1, [C_42_H_82_NO_8_P + H]^+^, being present in the whole tissue section but enriched in the cyst walls. Scale bars: 200 μm.

Additionally, Figure 2 shows that some of the lipids and metabolites varied not only in signal intensities but also in lateral distribution. For example, the red ion channel was chosen to represent an ion mainly found inside the cysts, while the green ion channel was chosen to represent an ion mainly found in the cyst walls or the outer part of the cysts. In contrast, the ion shown in blue was barely found inside the cysts, more reflecting the surrounding tissue. In total, the ion distribution patterns differed based on presence or absence and increase or depletion in the cyst center, the cyst walls, or the surrounding tissue. Some additional examples for positive- and negative-ion modes are shown in Figure 3.

Taking a deeper look into these ion signals reveals interesting findings. For example, the ion shown in Figure 3C was annotated as fructosebisphosphate (or other isomers, like glucosebisphosphate). It was found only inside the cysts and is an intermediate in glycolysis. Taubert et al. showed that *B. besnoiti* tachyzoite infection led to upregulation of glycolysis in host cells to fulfill the high energy needs of the replicating parasite.^52^ Even when bradyzoites are the slow-replicating stage of the parasite, it is interesting to see that they seem to perform glycolysis on their own, since the intermediate is not found in the host tissue but only in the parasitic tissue. Also, another intermediate of glycolysis, glucose phosphate, was found in the MS imaging experiments. This metabolite was found to be present in the whole tissue but with higher intensities inside the cysts.

The enrichment of the fatty acid (FA) 18:1 in the cyst walls (Figure 3F) and also FA 16:1 (not shown) can be a hint that these fatty acids are needed by the parasite for proliferation. However, the ions detected there can also be an artifact of the ionization process. We cannot exclude that they are fragmentation products, originating from phospholipids containing these fatty acid chains. Phospholipids in general are the main compounds of cell membranes, and parasitic cysts are mainly composed of one extremely enlarged host cell. Therefore, at least the outer parts of the cyst walls are composed of the former host cell membrane. On the other hand, FA 20:4 was mainly found in the surrounding tissue, but neither inside the cysts nor the cyst walls (see blue ion channel of negative-ion MS images in Figure 2).

### Ultrahigh-Resolution MS Imaging

**Figure 4:**
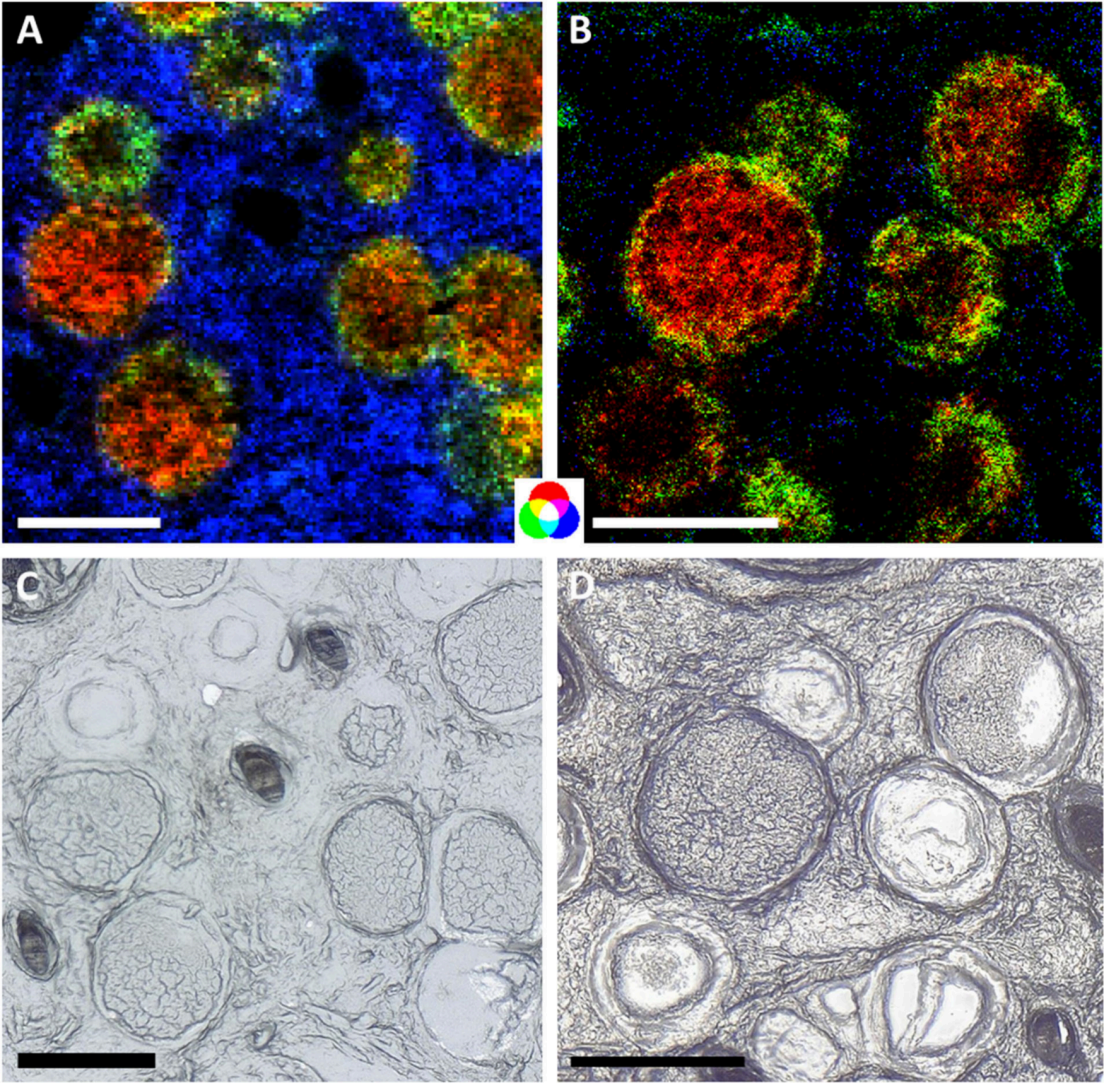
Improvement of the image quality when changing from 5 to 2 μm pixel size. *m*/*z* 732.5539, identified as PC 32:1 [C_40_H_78_NO_8_P + H]^+^, shown in red; *m*/*z* 798.5408, identified as PC 34:1 [C_42_H_82_NO_8_P + K]^+^, shown in green; and *m*/*z* 725.5566, annotated as SM 34:1 [C_39_H_79_N_2_O_6_P + Na]^+^, shown in blue. (A) *B. besnoiti*-infected skin tissue, measured with a 5 μm pixel size. (B) *B. besnoiti*-infected skin tissue, measured with 2 μm pixel size. The same ion channels were used to generate both images; however, signals are not adjusted to the same intensity scale. (C, D) Corresponding optical images. Scale bars are 200 μm.

Given the fact that, especially the cyst walls are thin structures of only a few micrometer thickness, we conducted ultrahigh-resolution MS imaging experiments on infected tissue samples. One example is shown in Figure 4. The left-hand side (A) shows infected tissue measured with 5 μm pixel size, and the right-hand side (B) shows a neighboring section measured with 2 μm pixel size. Both images show the same ions with the same color code. We did not find any additional metabolites specifically located in the cyst walls using a higher lateral resolution. This might be due to the lower signal intensities resulting from the smaller ablation spot size. Observed signal intensities were 20 to 40 times lower when reducing the laser spot area. Additionally, with smaller laser ablation spots, the total number of spots per analyzed area also increased. To visualize an appropriate area, we moved from 150 × 150 pixels (5 μm) to 300 × 300 pixels (2 μm). Therefore, the measurement time increased from 4 h (5 μm) to 15.5 h (2 μm) per sample. Also, we were not able to differentiate further between the different layers of the cyst walls. However, we were still able to detect known signals with sufficient signal intensities. As can be seen, the resulting images have a higher image quality in terms of structural details than the ones with 5 μm pixel size. Additionally, slight differences in ion distributions were found. While the ions of the red channel seem to be distributed nearly uniformly inside the cysts in Figure 4A, the higher resolution reveals slightly different distributions in Figure 4B. They do not seem to be uniformly spread but are more scattered. Looking at the corresponding optical image, the cysts seem to contain some cracks and might have shrunk during storage at −80 °C, resulting in inhomogeneous parasite and ion signal distributions.

The ultrahigh-resolution images better resolved the connecting area between the cyst content and the cyst wall. In Figure 4, the cyst content is mainly represented by the ion shown in red, while the cyst walls are represented by the ion shown in green. If both ions are found in one pixel, the overlap of the red and green ion channels results in a yellow pixel. In the less resolved ion image in Figure 4A (5 μm pixel size), many pixels on the cyst borders are yellow, indicating an overlap of green and red. In contrast, the ultrahigh-resolution ion image in Figure 4B (2 μm pixel size) contains only a few yellow pixels, while most pixels of the borders are clearly dedicated to either green or red.

Both filled and empty cysts are visible in the optical images. The empty cysts are thought to be preparational artifacts created during the cryosection process. We assume that only the outer cyst wall remained in the tissue section in these cases, and imperfect sample preparation leading to artifacts, in fact, gave us the opportunity to further study the composition of the walls. *B. besnoiti*-cyst walls are of high importance when it comes to studying new drug targets, since they are the connection between host and parasite stages (i.e., bradyzoites). One example, also found in the cyst walls of filled cysts but especially in the remainders of empty cysts, is the potassium adduct of PC(34:1), shown in green in Figure 4. Other examples for possible enrichment in the cyst walls can be found in Figure S4. One of these examples is the signal annotated as glucosyl ceramide (GlcCer) 41:6 in Figure S4B. Glucosyl ceramides as well as galactosyl ceramides are known to be present in the outer part of the lipid bilayer of cell walls,^53^ so it is reasonable to find them in the leftovers of a cyst.

### 3D Reconstruction

To gain deeper insights into the three-dimensional structures of the cysts inside the skin, two 3D reconstructions of *m*/*z* 798.5 and *m*/*z* 824.5 were created (Supporting Information Videos 1 and 2, respectively). This is especially helpful to examine the orientation of the cysts relative to each other. As already seen in the skin sections, the cysts seem to be grouped together. This is confirmed by the 3D view acquired from a set of 28 consecutive section measurements, each of them having a thickness and pixel size of 14 μm. Whether the cysts interact with each other has to be examined in further studies.

### Parasite Stage Comparison

The life cycle of *B. besnoitia* includes two different parasite stages in the intermediate host (for example, bovines). As such, during acute besnoitiosis, tachyzoite stages rapidly proliferate intracellularly, while the chronicity of infection is characterized by a slow replication process of bradyzoites within tissue cysts. Overall, it is well documented that apicomplexan tachyzoite and bradyzoite stages differ significantly in their antigenic^54,55^ and metabolic repertoire.^56,57^ Hence, the differential replication behavior of the parasite stages is also reflected in their metabolic activities. As such, *T. gondii* switches from aerobic respiration to mostly anaerobic metabolic pathways when converting from tachyzoites to bradyzoites.^56,57^

The current study revealed a total of 552 ion signals significantly changed in ion abundance in *B. besnoiti* cyst-infected skin tissue considering a mass range of *m*/*z* 250–1000. It is very difficult to isolate a sufficient number of bradyzoites from the cysts for MSI measurements. It relies on separation from the freshest skin samples directly after collection of the samples, which was impossible in this case. However, the conservation protocol used for the samples allowed us to analyze bradyzoites inside their cysts and within the host tissue. This is as close as possible to the *in vivo* situation and opened the opportunity to study host–parasite interactions in addition to the lipid profile of bradyzoites alone.

In a previous study, Kadesch et al. analyzed primary bovine endothelial host cell cultures infected with tachyzoite stages by AP-SMALDI MSI in a mass range from *m*/*z* 500 to 2000.^34^ They applied a pixel size of 10 μm and DHB as a matrix in positive-ion mode. Referring to these MSI data, we here compared changes driven by tachyzoite (Kadesch et al.) and bradyzoite (current study) infections. Overall, 82 ions that were found to be significantly changed in signal intensity in bradyzoite-containing skin measurements were also detected in tachyzoite-infected cell layers. Overall, there was no distinct lipid group found, being specific either for bradyzoite or tachyzoite infection. Many overlapping markers were found enriched in *B. besnoiti* cysts, suggesting that these compounds may either be needed for parasite development or metabolism and that related pathways may therefore be preserved. Interestingly, in negative-ion mode, there were also seven ions ((phosphatidylinositols (PI) PI(38:2), PI(38:4), PI(38:5), PE(38:4), PE-Cer(d44:1), CerP(d44:2), and PC(dO-36:4), all deprotonated) found in the tachyzoite measurements, present only outside the cysts or maybe in the cyst walls, but none found in the cyst content in the bradyzoite measurements.

All cyst-content-related markers in the *m*/*z* range of 500–1000 were also present in pure tachyzoites, except for the phosphatidylserine (PS) PS44:10. The almost perfect overlap between tachyzoite and bradyzoite infection-derived signals may result from the fact that the tachyzoite stage converts into the bradyzoite stage and vice versa; thus, the chemical composition of lipids and smaller metabolites may grossly be preserved. At least for *T. gondii*, tachyzoites and bradyzoites do not differ much in their structural composition;^58^ therefore, for *B. besnoiti* stages, molecular compositions should also be quite similar.

Overall, the most abundant lipid species found in *B. besnoiti* cyst-infected skin were PAs, PSs, PCs/PEs, TGs, PIs, and phopsphatidylglycerols (PGs) (see Figure S5). While little is known about *B. besnoiti*-mediated lipid requirements or compositions, data on *Plasmodium* erythrocyte infection also indicated elevated levels of PC, PE, and PA compared to noninfected cells.^51^ In *T. gondii* tachyzoites, PCs, PEs, PSs, and PIs were the most abundant phospholipids.^59^ Also, Welti et al. found PCs, PE-Cers, and PAs enriched in *T. gondii* compared to host cells.^60^ Additionally, Kadesch et al. mainly found PCs and PIs as infection markers for *T. gondii* and *B. besnoiti* tachyzoites,^34^ fitting well to our recent data.

## Conclusion

We were able to study the cyst-forming parasite *B. besnoiti* and its host in parallel using AP-SMALDI MSI. With database annotation and statistical analysis, we discovered 552 *B. besnoiti* infection-related ion signatures in the skin of cattle. The comparison of deduced MS images with corresponding light microscopic images allowed for the direct assignment of molecules to characteristic biological structures such as cyst compartments. Only minimal sample preparation had to be applied, enabling the analysis of delicate material, such as bradyzoites. In this study, we analyzed *B. besnoiti* cyst-infected skin tissue *ex vivo* and therefore addressed the chronic phase of disease with bradyzoite stages. To the best of our knowledge, this is the first *ex vivo* study of *B. besnoiti* bradyzoites using the MALDI MSI methodology. As expected, a plethora of metabolites matched previous data from *B. besnoiti* tachyzoite stages, which were obtained from *in vitro* cultures. Additionally, we employed ultrahigh-resolution MSI to further differentiate between biological structures. Also, we were able to identify some of the annotated ions by on-tissue MALDI MS/MS. Finally, we created a 3D reconstruction, showing the cysts inside the skin and their spatial orientation to each other.

Expanding the method to other substance classes and metabolites will further broaden our knowledge of the metabolism and composition of this neglected parasite.

In summary, we successfully implemented a novel AP-SMALDI MS-based method and applied it to a host–parasite tissue model. Further analyses are needed to elucidate the stage-specific substance requirements of this understudied parasite species.

### Limitations of the Study

Please note that most compounds were only annotated based on their accurate mass and thus might in fact be structural isomers with the same elemental composition. For unambiguous identification, liquid chromatography combined with tandem mass spectrometry from homogenized neighboring tissue sections is needed in future experiments, further supported by comparison to reference standards.

Validation of results from different individuals (*n* > 1) was not yet possible due to unavailability of material. We are convinced, however, that the generated data are nevertheless a useful basis for future research on *B. besnoiti*-infected skin.

## Supporting Information Available

Used chemicals, instrument settings, PCA and dendrogram of statistical analysis, additional MS images, and pie chart of found lipid species (PDF)
List of altered ions (XLSX)
Video 1 of 3D reconstruction, *m*/*z* 798.5 (MP4)
Video 2 of 3D reconstruction, *m*/*z* 824.5 (MP4)
