# Supplementary material for: Mass Spectrometry Imaging of Lipid and Metabolite Distributions in Cysts of Besnoitia besnoiti-Infected Bovine Skin
Source: J Am Soc Mass Spectrom. 2025 Apr 8;36(5):1017–26. doi: 10.1021/jasms.4c00466 (PMC12063159; doi:10.1021/jasms.4c00466)
Supplement: Supplementary file 1 — js4c00466_si_001.pdf [file js4c00466_si_001.pdf]

Mass spectrometry imaging of lipid and metabolite distributions in cysts of *Besnoitia besnoiti*-infected bovine skin

Katja R. Wiedemann[1], Stefanie Gerbig[1], Parviz Ghezellou[1], Alejandra Pilgram[1], Carlos Hermosilla[2], Anja Taubert[2], Liliana M. R. Silva[2,3,4*], Bernhard Spengler[1*]

[1]*Institute of Inorganic and Analytical Chemistry, Justus Liebig University Giessen, 35392 Giessen, Germany*
[2]*Institute of Parasitology, Justus Liebig University Giessen, 35392 Giessen, Germany*
[3]*Egas Moniz Center for Interdisciplinary Research (CiiEM); Egas Moniz School of Health & Science, 2829-511 Caparica, Almada, Portugal*
[4]*MED – Mediterranean Institute for Agriculture, Environment and Development & CHANGE – Global Change and Sustainability Institute, Universidade de Évora, 7006-554 Évora, Portugal*

* Authors to whom correspondence should be addressed, these authors contributed equally
E-Mail addresses: bernhard.spengler@anorg.chemie.uni-giessen.de; liliana.silva@vetmed.uni-giessen.de

Supporting information

Chemicals

Table S1: Used chemicals and their suppliers.

Chemical name	Quality grade	manufacturer
1,5-diaminonaphthalene	97%	Thermo Fisher, Kandel, Germany
2,5-dihydroxybenzoic acid	for synthesis	Merck, Darmstadt, Germany
acetone	HiPerSolv	VWR International, Fontenay-sous-Bois, France
Eosin Y solution		Sigma-Aldrich, Steinheim, Germany
ethanol	Uvasol	Merck, Darmstadt, Germany
Eukitt quick hardening medium		Sigma-Aldrich, Steinheim, Germany
gelatin		VWR International, Leuven, Berlgium
Mayer's hematoxylin solution		Sigma-Aldrich, Steinheim, Germany
methanol	Rotisolv HPLC gradient grade	Carl Roth, Karlsruhe, Germany
trifluoro acetic acid	Uvasol	Merck, Darmstadt, Germany
water	HiPerSolv	VWR International, Fontenay-sous-Bois, France
xylene	for analysis	Merck, Darmstadt, Germany

Table S2: Settings used for data acquisition with MALDI MSI.

Parameter	Setting
m/z	250-1000
Resolution	240,000 at *m/z* 200
Ion injection time	500 ms
Scan rate	1.6 pixel / s
Spray voltage	3 kV
Capillary temperature	250°C
Lock mass	*m/z* 585.06396 [4 DHB − 3 H_2O + Na]$^+$ in positive-ion mode; *m/z* 313.14587 [2DAN − H − H_2]$^-$ in negative-ion mode
Calibration	Positive-ion mode:<br>*m/z* 273.03936 [2 DHB + H -2 H_2O]$^+$<br>*m/z* 409.05541 [3 DHB + H – 3 H_2O]$^+$<br>*m/z* 545.07145 [4 DHB + H – 4 H_2O]$^+$<br>*m/z* 681.08750 [5 DHB + H – 5 H_2O]$^+$<br>*m/z* 817.10354 [6 DHB + H – 6 H_20]$^+$<br>*m/z* 953.11958 [7 DHB + H – 7 H_2O]$^+$<br>Negative-ion mode:<br>*m/z* 289.03538 [2 DHB – H – H_20]$^-$<br>*m/z* 329.02789 [2 DHB – 2 H + Na]$^-$<br>*m/z* 465.04393 [3 DHB – 2 H + Na – H_2O]$^-$<br>*m/z* 579.07803 [4 DHB – H- 2 H_2O]$^-$<br>*m/z* 715.09407 [5 DHB – H – 3 H_2O]$^-$
Pixel size	5 µm
Mode	2D pixel mode

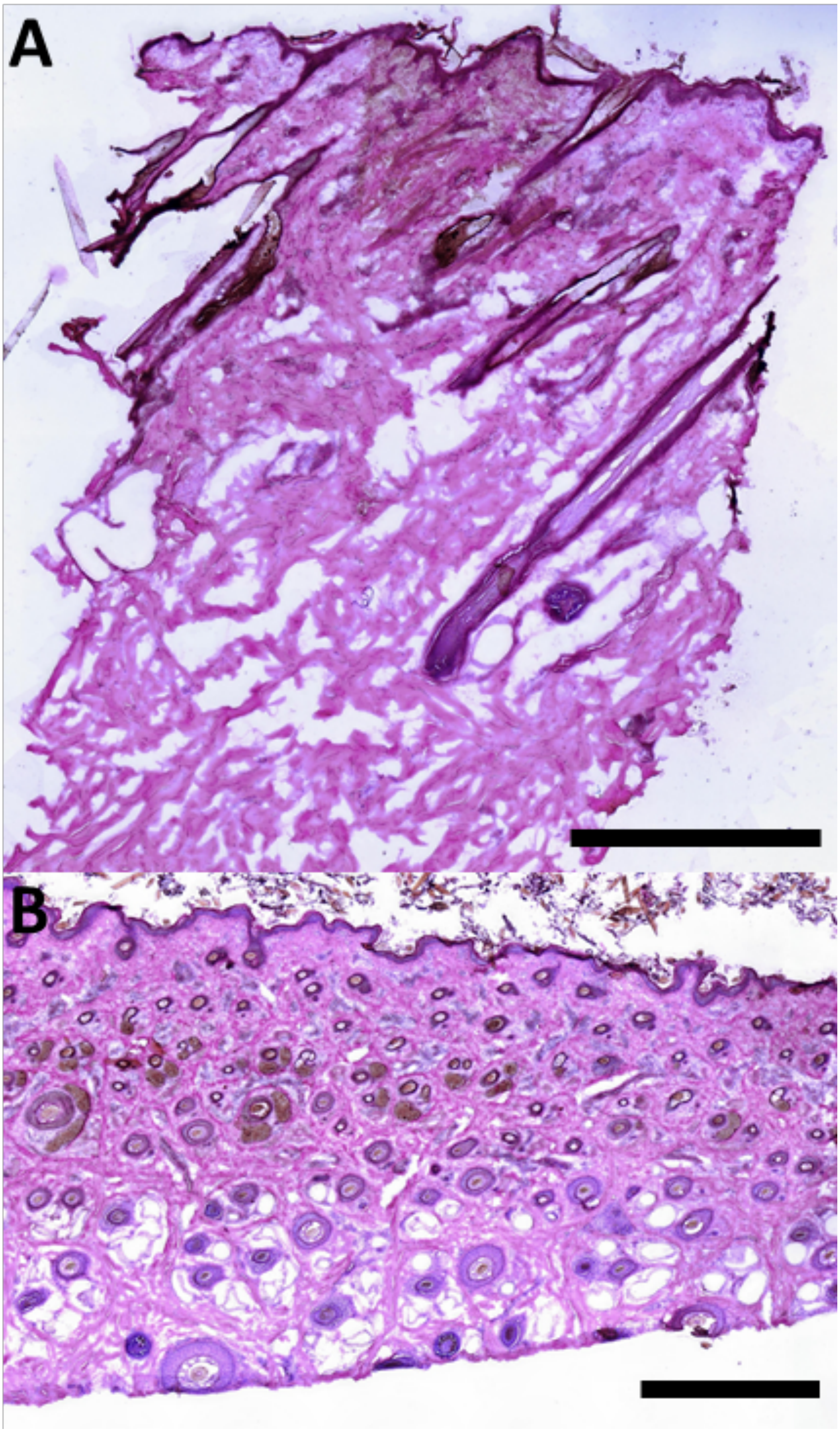

Figure S1: Representative H&E-stained tissue sections of the two different control animals. Both showed neither clinical symptoms nor tissue cysts. Scale bars are 1 mm.

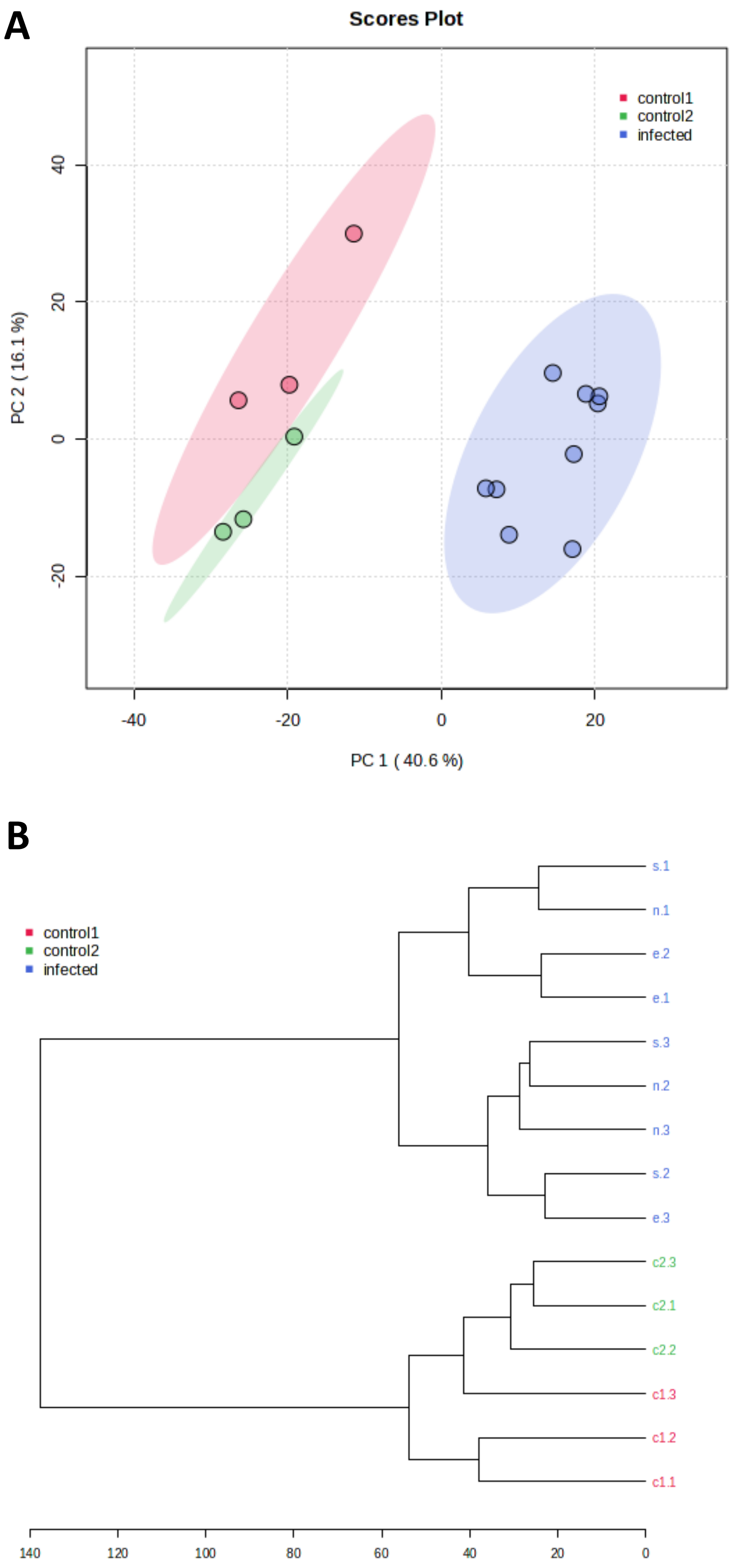

Figure S2: Results of statistical analyses. PCA (A) shows a clear separation of control samples and infected ones. Additionally, control samples cluster together, even when originating from different animals. This is also shown in the dendrogram (B).

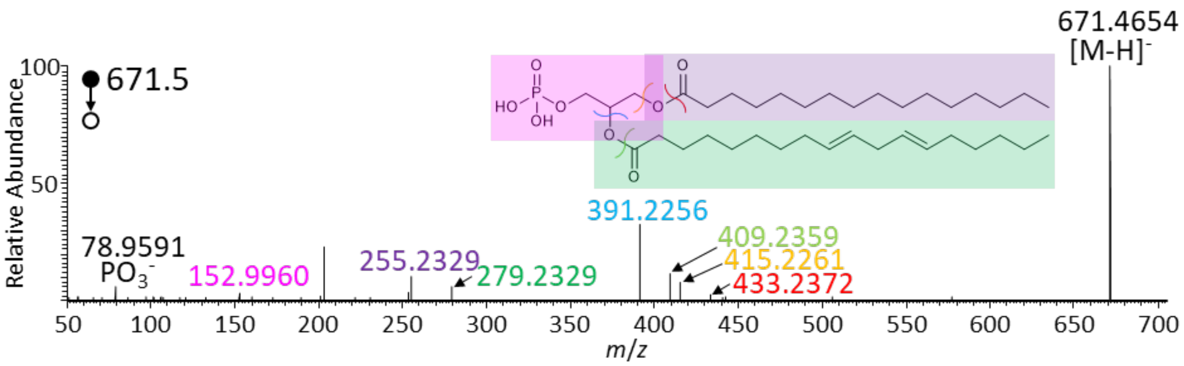

Figure S3: On-tissue tandem mass spectrum of *m/z* 671.4654, fragmented with a normalized collision energy of 20 and identified as [PA(16:0_18:2)-H]-.

Table S3: Annotation of fragment ions in spectrum Figure 4.

Mass detected	Fragment ion	Theoretical mass	Deviation / ppm	Fragment
671.4654	$[C_{37}H_{69}PO_8\text{-}H]^-$	671.4652	+ 0.30	Precursor ion
433.2372	$C_{21}H_{38}PO_7^-$	433.2355	+ 3.92	Loss of FA 16:0 chain as ketene
415.2261	$C_{21}H_{36}PO_6^-$	415.2250	+ 2,65	Loss of FA 16:0
409.2359	$C_{19}H_{38}PO_7^-$	409.2355	+ 0,98	Loss of FA 18:2 chain as ketene
391.2256	$C_{19}H_{36}PO_6^-$	391.2250	+ 1,53	Loss of FA 18:2
279.2329	$[C_{18}H_{32}O_2\text{-}H]^-$	279.2324	+ 1,79	FA 18:2
255.2329	$[C_{16}H_{32}O_2\text{-}H]^-$	255.2324	+ 1,96	FA 16:0
152.9960	$C_3H_6PO_5^-$	152.9953	+ 4,58	PA head group $-H_2O$
78.9591	PO_3^-	78.9585	+ 7,60	PO_3^-

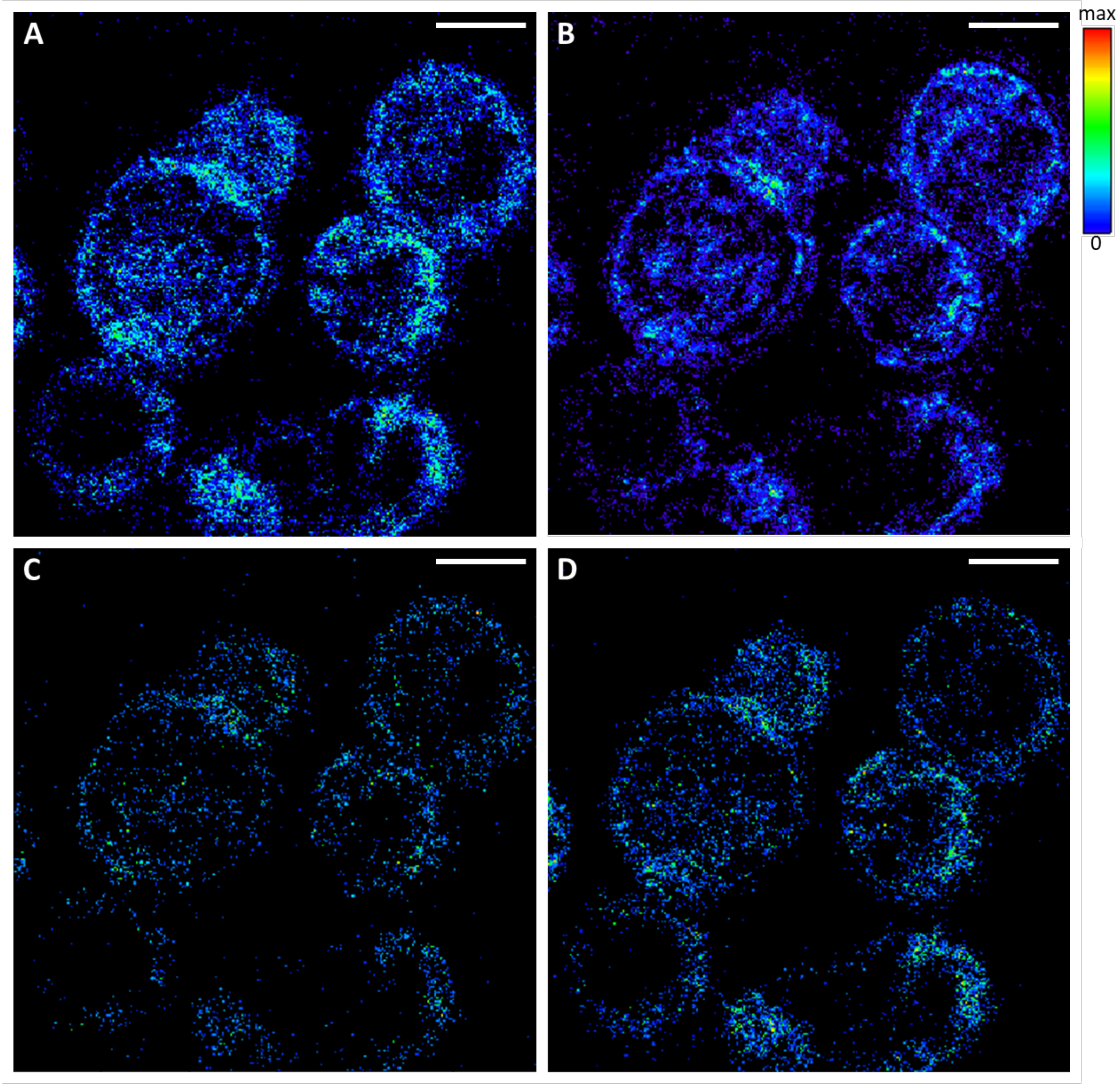

Figure S4: Examples of lipids mainly found inside the skin cysts with possible enrichment in the cyst walls. A) *m/z* 744.4942, identified as PC 30:0 [$C_{38}H_{76}NO_8P$ + K]$^+$. B) *m/z* 842.5543, annotated as GlcCer 41:6 [$C_{47}H_{81}NO_9$ + K]$^+$. C) *m/z* 728.5202, identified as PC 32:3 [$C_{40}H_{74}NO_8P$ + H]$^+$. D) *m/z* 716.4628, annotated as PC 28:0 [$C_{36}H_{72}NO_8P$ + K]$^+$. Imaging experiments were performed with 2 µm pixel size, scale bars are 100 µm. Please note that for clearness only the most probable annotation was mentioned here. All possible annotations can be found in the supplementary excel sheet ("altered ions.xlsx"). However, PC 30:0 and PC 32:3, shown in A and C, respectively, were identified by on-tissue MS/MS.

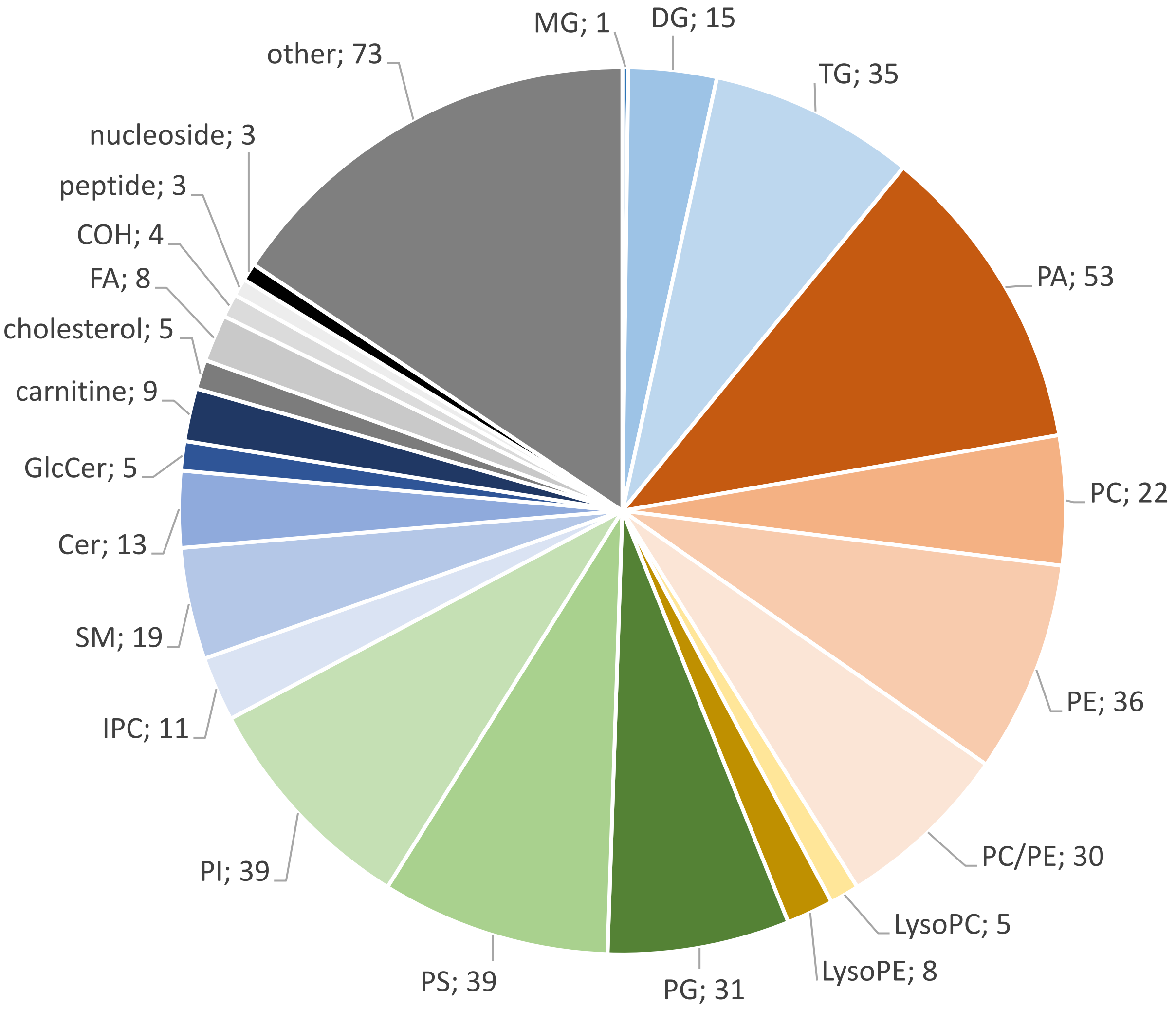

Figure S5: Substance class distribution of signals significantly changed in intensities in *B. besnoiti*-infected skin (substance class; number of species). Only one adduct was considered per species, so that species were not counted multiple times when they occurred as different adducts. MG: monoacylglycerol, DG: diacylglycerol, TG: triacylglycerol, PA: phosphatidic acid, PC: phosphatidylcholine, PE: phosphatidylethanolamine, PG: phosphatidylglycerol, PS: phosphatidylserine, PI: phosphatidylinositol, IPC: ceramide phosphoinositol, SM: sphingomyelin, Cer: ceramide, GlcCer: glucosylceramide, FA: fatty acid, COH: carbohydrate.
